# Two founder mutations in the *SEC23B* gene account for the relatively high frequency of CDA II in the Italian population

**DOI:** 10.1002/ajh.22096

**Published:** 2011-06-14

**Authors:** Roberta Russo, Antonella Gambale, Maria Rosaria Esposito, Maria Luisa Serra, Annaelena Troiano, Ilaria De Maggio, Mario Capasso, Lucio Luzzatto, Jean Delaunay, Hannah Tamary, Achille Iolascon

**Affiliations:** 1 CEINGE Biotecnologie AvanzateNapoli, Italy; 2 Department of Biochemistry and Medical Biotechnologies, University of Naples Federico IINapoli, Italy; 3 Department of Biomedical Sciences and Biotechnologies, University of Study of CagliariCagliari, Italy; 4 Istituto Toscano TumoriFlorence, Italy; 5 UMR_S 779, INSERM, Faculté de Médecine Paris-Sud, Université Paris-Sud94275 Le Kremlin-Bicêtre, Paris, France; 6 Pediatric Hematology/Oncology Division, Schneider Children's Medical Center of Israel and Sackler Faculty of Medicine, Tel-Aviv UniversityTel-Aviv, Israel

## Abstract

Congenital Dyserythropoietic Anemia type II is an autosomal recessive disorder characterized by unique abnormalities in the differentiation of cells of the erythroid lineage. The vast majority of CDA II cases result from mutations in the *SEC23B* gene. To date, 53 different causative mutations have been reported in 86 unrelated cases (from the CDA II European Registry), 47 of them Italian. We have now identified *SEC23B* mutations in 23 additional patients, 17 Italians and 6 non-Italian Europeans. The relative allelic frequency of the mutations was then reassessed in a total of 64 Italian and 45 non-Italian unrelated patients. Two mutations, E109K and R14W, account for over one-half of the cases of CDA II in Italy. Whereas the relative frequency of E109K is similar in Italy and in the rest of Europe (and is also prevalent in Moroccan Jews), the relative frequency of R14W is significantly higher in Italy (26.3% vs. 10.7%). By haplotype analysis we demonstrated that both are founder mutations in the Italian population. By using the DMLE+ program our estimate for the age of the E109K mutation in Italian population is ≈2,200 years; whereas for the R14W mutation it is ≈3,000 years. We hypothesize that E109K may have originated in the Middle East and may have spread in the heyday of the Roman Empire. Instead, R14W may have originated in Southern Italy. The relatively high frequency of the R14W mutation may account for the known increased prevalence of CDA II in Italy. Am. J. Hematol. 86:727–732, 2011. © 2011 Wiley-Liss, Inc.

## Introduction

Congenital dyserythropoietic anemia (CDA) was first described in 1968 as a condition characterized by a paradoxical association of anemia and reticulocytopenia with erythroid hyperplasia in the bone marrow [1, 2]. It soon became clear that the condition was heterogeneous, and three forms became well known [1], with Type II being the most frequent. The prevalence of CDAs in Europe has been recently assessed. The combined prevalence of CDA I and CDA II (based on all cases reported in the last 42 years) has the highest value in Italy (2.49/million). CDA II (367 cases) is relatively more frequent than CDA I (122 cases), with an overall ratio of approximately 3.0 [3].

CDA II is an autosomal recessive condition presenting with moderate to severe normocytic or microcytic anemia, with a normal or insufficiently increased reticulocyte count, chronic or intermittent jaundice, splenomegaly [4]. Bone marrow of CDA II patients is characterized by presence of bi-nucleated or multinucleated normoblasts. In addition, upon electron microscopy, vesicles of endoplasmic reticulum appear to be running beneath the plasma membrane [5]. Furthermore a number of abnormalities affecting glycosylation and/or levels of erythrocyte glycoconjugates were observed. Hypoglycosylation of erythrocyte anion exchanger 1 represents a key for the diagnosis [6] and suggests a defect in vesicles trafficking.

After the demonstration that 28 unrelated cases of CDA II were associated with mutations in the *SEC23B* gene [7], a total of 53 different causative mutations have been identified in 86 unrelated cases, mostly of European origin [7–11]. The *SEC23B* gene encodes the SEC23B component of the COPII complex, involved in the anterograde transport of correctly folded protein from the endoplasmic reticulum towards the Golgi [12]. Although most of the mutations found in *SEC23B* gene appear to result from independent events, 4 mutations (R14W, E109K, R497C, I318T) account for more than 50% of mutant alleles, which is a help with respect to molecular diagnosis [9, 11]. In a recent paper, Amir and colleagues found that in Israel all patients diagnosed with CDA II to date are of North-African descent, mainly Moroccan-Jews, and they are all homozygotes for the E109K mutation. Moreover, the authors have observed in these patients a common haplotype, suggesting a founder mutation, estimated to have taken place about 2,400 years ago [13].

Here, we report on 23 additional patients, 17 Italian, and 6 non-Italian Europeans, and we show that E109K and R14W account for about 54% of all patients in Italy. By extensive haplotype analysis we show that the recurrent *SEC23B*-R14W mutation found in most of the Italian families with CDA II is likely due to a founder effect, with the founder mutation having occurred probably in Southern Italy. By contrast, E109K is more widespread within Europe.

## Design and Methods

*Subjects and relative allelic frequency assessment*. To date, 86 unrelated european cases of CDA II due to 53 different causative mutations have been described [7–11]. Among them, 47 unrelated cases had Italian origin with 31 different mutations, 39 were non-Italian European (NIE) patients with 32 different mutations. Furthermore we included 17 Italian and 6 NIE unrelated cases still unpublished, with an overall count of 64 Italian and 45 NIE patients (Supporting Information Table Is).

Haplotype analysis was carried out in 31 Italian CDA II patients from 23 independent families and six NIE cases from four unrelated families. Moreover, we confirmed the E109K haplotype on three Moroccan Jewish patients. We also enrolled 47 healthy subjects originated from Italy (Supporting Information Table IIs). Blood samples were collected following approval by the local ethics committees. Written consent was obtained from all patients in accordance with the Declaration of Helsinki.

*SEC23B sequencing*. Genomic DNA was prepared from peripheral blood using Wizard Genomic DNA purification kit (Promega, Milano, Italy). Mutational search, oligonucleotide primers design and direct sequencing were performed as previously described [9]. *SEC23B* sequence primers are available upon request (achille.iolascon@unina.it). Nucleotide numbering reflects cDNA numbering with +1 corresponding to the A of ATG translation initiation codon in the reference sequence (Ensembl transcript ID: ENST00000377475).

*Marker selection*. Single nucleotide polymorphisms (SNPs) were selected from the HapMap Project Home Page (http://hapmap. ncbi.nlm.nih.gov/). By using the International HapMap Project phase II [14] we downloaded a list of 9370 SNPs within a region of 1,685 kb upstream, 2,656 kb downstream of the *SEC23B* gene. From this list, all SNPs with a minor allele frequency (MAF) > 0.35 were chosen to identify the tag-SNPs, to avoid redundant typing of sets of variants that are in complete linkage disequilibrium with each other [15].

By using Haploview software package [16], 12 tag-SNPs with a threshold of r^2^ > 0.8 were selected (rs241141, rs8121302, rs6111826, rs761463, rs6136363, rs13039328, rs6045524, rs6132097, rs6045592, rs742731, rs6105992, rs6045803) covering a region of about 1.2 Mb.

*SNPs genotyping*. SNPs genotyping was performed with direct sequencing. All fragments were amplified from genomic DNA by PCR in a 25 μl volume with Expand High Fidelity PCR System, following manufacturer's protocol (Roche, Germany). The oligonucleotide primers were designed from the sequence downloaded from the NCBI dbSNP Home Page (http://www.ncbi.nlm.nih.gov/projects/SNP/), by Primer3 program (Primer3 v. 0.4.0, freeware online) (Supporting Information Table IIs). The PCR products were checked by DNA agarose gel electrophoresis. Direct sequencing was performed using the BigDye® Terminator Cycle Sequencing Kit (Applied Biosystems, Branchberg, NJ) and a 3730 DNA Analyzer (Applied Biosystems).

*Haplotype analysis*. For each SNP, the allele frequencies were defined, and testing for Hardy-Weinberg equilibrium was performed in a control sample from the general population of Italy (Supporting Information Table IIs). Haplotypes at the gene locus were defined using the modified estimation-maximization algorithm implemented in the Haploview software package [16]. We included genotypes information of 47 Italian unrelated controls and 174 Caucasian controls (HapMap CEU), downloaded from the HapMap Project home page. When we compared the allele and genotype frequencies of HapMap CEU population with those of 47 Italian healthy subjects, we did not find any significant difference (Supporting Information Table IIs).

*Age of mutation analysis*. DMLE+ version 2.3 developed by Reeve and Rannala (http://dmle.org/) was used to estimate the age of both, R14W and E109K mutations. This program was designed for high-resolution mapping of a disease mutation and estimation of its age. The method is based on the observed linkage disequilibrium between a disease mutation and linked markers in DNA samples of unrelated normal individuals and affected patients. The program uses the Markov Chain Monte Carlo algorithm to allow Bayesian estimation of the mutation age based on the following parameters: the observed haplotypes (or genotypes) in samples of unrelated normal or affected chromosomes, map distances between markers, the position of the mutation relative to the markers and the population growth rate, and an estimate for the proportion of disease-bearing chromosomes [17]. We performed two analyses: (i) age estimation of R14W haplotypes of 23 italian patients; (ii) age estimation of E109K haplotypes of eight italian cases.

The population growth rate (*r*) was estimated by the equation, accordingly to previously described method [18]: *T*_1_ = *T*_0_e^(gr)^, in which *T*_1_ is the estimated size of population today in Italy (58 million), *T*_0_ is the estimated size of the ancestral population (2 million between the 5th and 3rd centuries BC), and g is the number of generations between these 2 time points (*g* = 64.4 considering 25 years for a generation). Accordingly, population growth rate was estimated to be approximately equal to 0.05. We used a proportion of population sampled of 0.0072 for E109K and 0.0045 for R14W cases, using a previously described method [19].

## Results

### Sequencing analysis and frequency of individual *SEC23B* mutations

We tested for mutations in the *SEC23B* 23 unpublished unrelated cases (Supporting Information Table Is). The vast majority of them had two mutations (in the homozygous or compound heterozygous state), in accordance with the pattern of autosomal recessive inheritance. Five patients (F53P1, F62P1, F64P1, F65P1, F66P1) of the 23 studied showed only one mutation in the heterozygous state (Supporting Information Table Is). By combining the data with those previously available we have a total of 64 subjects from Italy and 45 NIE. Overall we found 31 different causative mutations in Italian patients. They included 14 missense, eight nonsense, four frameshift, four splice site mutations, and only one amino acid deletion. The distribution of different types of mutations is similar in NIE cases (19 missense, eight nonsense, three frameshift, two splicing).

**Figure 1:**
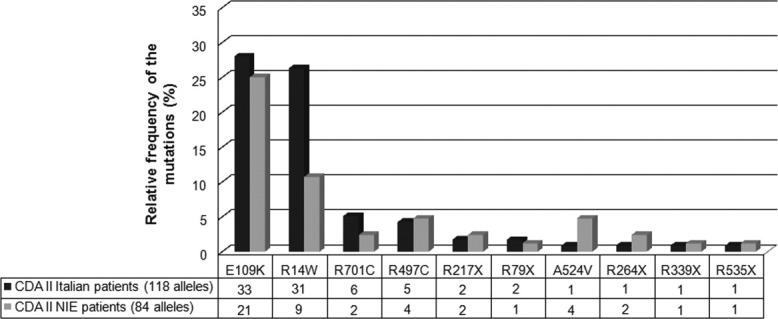
Allelic frequency of the mutations. The chart shows the results of the analysis performed on 10 mutations (E109K, R14W, R701C, R497C, R217X, R79X, A524V, R264X, R339X, R535X) shared between two cohorts. The allelic frequency has been assessed on 118 alleles from Italian cases and 84 from those not Italian.

In agreement with previous findings [9], the commonest mutations in the Italian cases were the missense substitutions E109K and R14W (28.0% and 26.3% respectively). When comparing the relative frequencies of 10 mutations (Fig. 1) present in both Italian and NIE cases, R14W stood out as far more common in Italian CDA II patients than in NIE (26.3% vs. 10.7%). By contrast, E109K had about the same frequency in the two groups (28.0% in Italian patients vs. 25.0% in NIE) (Fig. 1).

### Haplotype analysis

**Figure 2:**
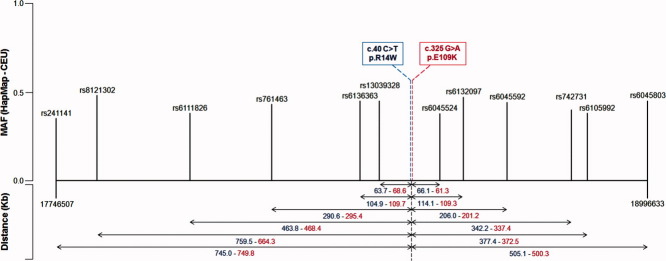
SNP markers localization upstream and downstream E109K and R14W mutations. The chromosomal region containing the SEC23B gene is shown. Arrows indicate the distances of SNP markers from the location of the SEC23B mutation. Names of SNP markers are shown on the bars, whose height is shown to scale with the MAF. Distances in kb of SNP markers from both mutations, E109K and R14W, are highlighted in gray and black, respectively. [Color figure can be viewed in the online issue, which is available at wileyonlinelibrary.com.]

In order to investigate the evolution of CDA II in Italy we used the haplotype method in a case-control study [20], by selecting SNP markers located near the commonest *SEC23B* mutations, R14W and E109K. Specifically, we examined 12 tag-SNP markers within 1.2 Mb within the *SEC23B* gene locus (Fig. 2). All of 23 Italian R14W patients from 16 independent families were heterozygous for this mutation (Table I, Supporting Information Fig. 1s). Of these R14W-patients, 47.2% shared a common haplotype (CACACCGC), composed by 8 SNPs (rs761463, rs6136363, rs13039328, rs6045524, rs6132097, rs6045592, rs742731, rs6105992) spanning a 667.9 kb region upstream and downstream the mutation (Table I). The same haplotype was found only in a small percentage of Italian controls (4%) and in HapMap CEU population (1%).

**I:** Haplotypes Flanking the R14W Mutation in 23 Italian Patients

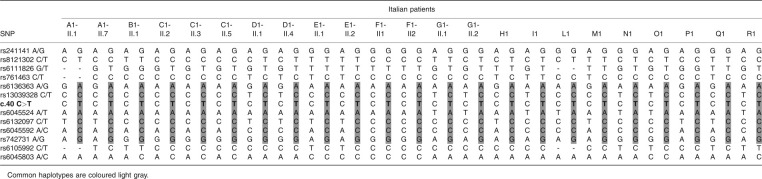

For the E109K mutation we had 14 homozygotes and 2 heterozygotes (Table II, Supporting Information Fig. 2s). Haplotype analysis showed that E109K-patients carried a common haplotype (CATAGT, 96.4%) composed by 6 SNPs (rs13039328, rs6045524, rs6132097, rs6045592, rs742731, rs6105992) spanning a 441.1 kb region within *SEC23B* upstream and downstream the mutation (Table II). The same haplotype was found only in a small percentage in Italian controls (5%) and in HapMap CEU population (3%). The same haplotype analysis in 3 Moroccan Jewish patients, homozygous for the E109K mutation, showed a similar chromosomal background, except for one marker (rs6105992) not conserved in the haplotype of the Moroccan Jewish patients (Supporting Information Table IIIs).

**Table II:** Haplotypes Flanking the E109K Mutation in Eight Italian Patients and 6 non-Italian European Patients

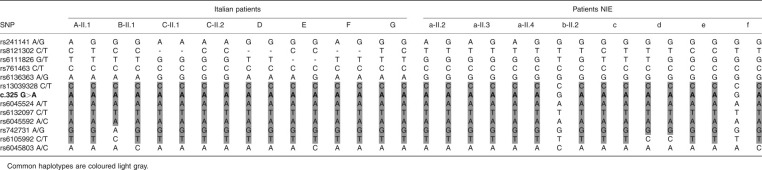

### Time of origin of the R14W and E109K mutations

**Figure 3:**
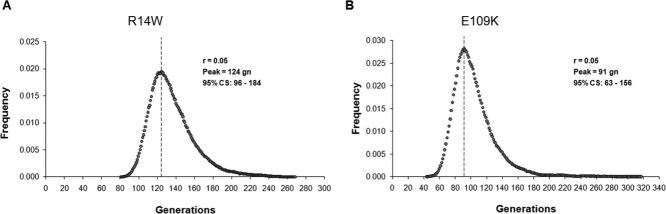
R14W and E109K age estimation. Age estimation of SEC23B R14W (A) and E109K (B) mutations. The posterior probability plots of the mutations age (in generations), as estimated by the software DMLE+2.3. The analyses were performed according to growth rate (*r*) of Italian populations. The vertical lines represent the average age values that had the highest probability (peak). The average 95% credible set (CS) of values for each posterior density is shown.

We used the DMLE+ program to obtain a Bayesian estimate of how old these mutations might be. On the basis of 23 R14W Italian patients we obtained a peak at 124 generations (95% credible set, 96–184) (Fig. 3A). Therefore, if the generation span is considered to be 25 years [21], this mutation would date back approximately to 3,000 years ago.

For E109K the same analysis revealed an estimated age of 91 generations (95% credible set, 63–156), calculated using haplotypes frequencies of the 8 Italian patients (Fig. 3B). Thus, this mutation would date back approximately to 2,200 years ago.

## Discussion

CDA II results from mutations that cause loss of function of the *SEC23B* gene. As for other autosomal recessive conditions, it is not surprising that many different mutations are found in patients, because there are many amino acid changes that can produce loss of function. To date we know 53 different mutations in *SEC23B* causing CDA II [7–11]. Here, we expanded the cohort of CDA II patients of European Registry (109 CDA II cases) including 17 Italian and 6 NIE unrelated cases still unpublished.

In this study, we compared the relative allelic frequency of *SEC23B* mutations in two cohorts of cases, 64 Italian and 45 NIE cases. We show (Fig. 1) that, whereas the majority of *SEC23B* mutations are found only occasionally, two mutations, R14W and E109K are relatively common in both cohorts, substantially confirming our previous data [9, 11]. Nevertheless, R14W variant showed a higher recurrence in Italian CDA II patients when compared to NIE patients (26.3% vs 10.7%), while E109K substitution showed almost the same allelic frequency between both groups (28.0% in Italian and 25.0% in NIE). In general, there are two possible explanations. Either (a) the mutation has been positively selected, or (b) it has spread by genetic drift: *i.e*., it results from a so-called founder effect. It is difficult to imagine that mutations that give no known phenotype in the heterozygous state, and cause a disease in the homozygous state, can be positively selected; therefore in principle (b) seems more likely in this case. A founder effect implies that identical mutant genes we see today have a single ancestral origin; and the spread of the mutant gene may have been greatly favored if at some stage a small population in which the gene was present has undergone rapid expansion. Haplotype analysis can provide minimum estimates for the time of origin of a founder mutation; more exactly, of the time when the mutation has spread, presumably because of population bottleneck was followed by expansion.

**Figure 4:**
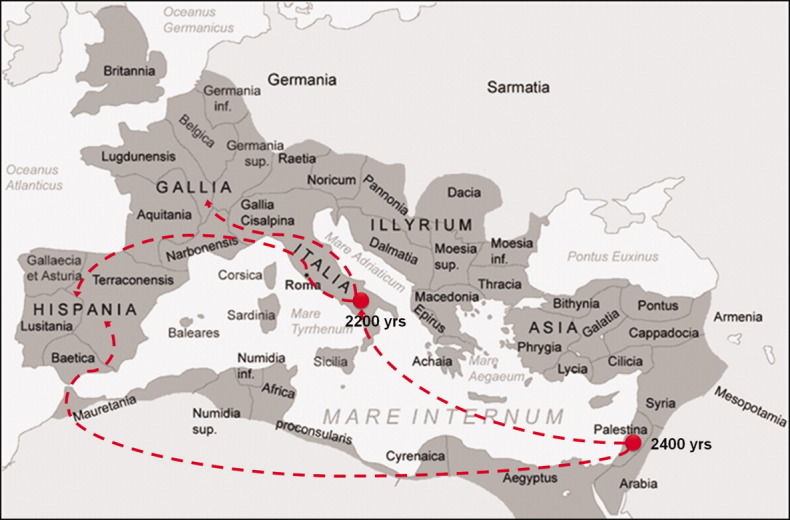
Dissemination of the E109K mutation in the Mediterranean area. The map shows the empire's greatest and largest territory in the year of 116 AD, around the time of Caesar Augustus (63 BC–14 AD) [The map is modified from a picture covered by Copyright AskRickToday.Com]. The dashed black arrows indicate the hypothetical migratory movements that led to the spread of the mutation in the Mediterranean area. Dots indicate the geographical areas in which the E109K mutation first occurred. [Color figure can be viewed in the online issue, which is available at wileyonlinelibrary.com.]

As we already stated, E109K and R14W mutations occurred in codons containing a complete or overlapping CpG dinucleotide (gc***cG***AAttg and gaa***CG***Ggat, respectively), a “hot spot” for mutations [9]. Nevertheless, our analysis strongly supports the notion that E109K and R14W are both examples of founder effects. E109K is particularly common in Moroccan-Jewish patients with CDA II [13], but it is also common in Italy and in other parts of Europe. We have now found that three Moroccan-Jewish E109K-patients have a chromosomal background similar to those found in Italian and European patients. Considering the wide confidence limits of any estimate of the origin of a mutation, our result of 2,200 years are not far from the estimate of 2,400 years given by Amir [13]. Alternatively, these different estimates might reflect several successive waves of expansion. We could hypothesize that the mutation was born in the Middle Est 2,400 years and arrived in European regions approximately at the time of Caesar Augustus when Roman Empire had the maximum expansion: thus, this mutation may then have spread throughout the Mediterranean area and perhaps elsewhere in the Roman Empire (Fig. 4).

For the most frequent mutation in Italian CDA II patients, R14W, we found a common haplotype (CACACCGC) in 47% of 23 heterozygote Italian patients: of note, this haplotype is different from those observed in E109K-patients. We estimated that this mutation would be originated approximately 3000 years ago, at the time when much of Southern Italy was a Greek colony, the *Magna Graecia*. On the basis of the geographic distribution of CDA II, showing a concentration of this disease in Southern Italy and in Mediterranean countries, we had previously suggested that a particular CDA II mutation arose or was introduced in Southern Italy, from where it might have spread over the rest of the country [4]. Our new data fully support this suggestion: of 16 unrelated R14W-patients here analyzed, 13 (81%) were from Central and Southern Italy. In a recent review of epidemiologic data it was confirmed that in Italy the prevalence of CDA II is higher than in other European countries, ∼2.49 cases per million [3]. If we subtract from this figure the contribution of patients with the R14W mutation, we find that the prevalence in Italy would fall in line with the rest of Europe: this means that the epidemiological anomaly of CDA II in Italy is accounted by this particular founder effect.

In this study, we characterized the allelic distribution of *SEC23B* gene mutations found in CDA II Italian patients, compared to those found in not Italian European cases. We demonstrated that the most frequent amino acid substitutions, R14W and E109K, are founder mutations in the Italian population; but, the first one may have originated in Southern Italy (3,000 years ago), while the latter is more widespread within Europe.
