# Supplementary material for: Two founder mutations in the SEC23B gene account for the relatively high frequency of CDA II in the Italian population
Source: Am J Hematol. 2011 Jun 14;86(9):727–32. doi: 10.1002/ajh.22096 (PMC3258542; doi:10.1002/ajh.22096)
Supplement: Supplementary file 1 [file ajh0086-0727-SD1.doc]

**Supplemental material**

| **Table Is. CDA II patients and *SEC23B* mutations** | | | | | |
| --- | --- | --- | --- | --- | --- |
| **Number of unrelated cases** | **Patient ID** | **Gender** (m)ale or (f)emale | **Geigraphical origin** | ***SEC23B* causative mutations** (exon number) | |
|  |  |  |  | *Allele 1* | *Allele 2* |
| 1 | F50P1 | f | Italian | E109K (4) | E109K (4) |
| 2 | F51P1 | f | Italian | R14W (2) | H608IfsX7 (16) |
| 3 | F52P1 F52P2 | f; f | Italian | R14W (2) | R701C (18) |
| 4 | F53P1 | f | Italian | I318T (8) | - |
| 5 | F54P1 | f | Italian | R14W (2) | R550X (14) |
| 6 | F55P1 | m | Italian | R14W (2) | c.689+1G>A (6) |
| 7 | F56P1 | f | Italian | E109K (4) | E109K (4) |
| 8 | F57P1 | f | Italian | R14W (2) | R217X (6) |
| 9 | F58P1 | f | Italian | R14W (2) | R701C (18) |
| 10 | F59P1 | f | Italian | E109K (4) | E109K (4) |
| 11 | F60P1 | f | Italian | R14W (2) | R264X (7) |
| 12 | F61P1 F61P1 | f; f | Italian | R14W (2) | H607HfsX7 (16) |
| 13 | F62P1 | m | Italian | E109K (4) | - |
| 14 | F63P1 | f | Italian | R14W (2) | c.689+1G>A (6) |
| 15 | F64P1 | f | Italian | E109K (4) | - |
| 16 | F65P1 | f | Italian | R550X (14) | - |
| 17 | F66P1 | m | Italian | R701C (18) | - |
| 18 | F67P1 | m | NIE | R18H (2) | A524V (14) |
| 19 | F68P1 | f | NIE | Y462C (12) | Y462C (12) |
| 20 | F69P1 | m | NIE | E109K (4) | D239G (7) |
| 21 | F70P1 | m | NIE | R14W (2) | R554X (14) |
| 22 | F71P1 | m | NIE | R14W (2) | R324X (8) |
| 23 | F72P1 | m | NIE | R313H (8) | R313H (8) |

NIE, Non-Italian European

| **Table IIs. Allele and genotype frequencies in healthy controls** | | | | | | | | | | | | |
| --- | --- | --- | --- | --- | --- | --- | --- | --- | --- | --- | --- | --- |
| **refSNP Cluster Report** | **RefSNP Alleles** | **HapMart CEU controls** (N=174)^‡^ | | | | | **Healthy subjects from Italy** (N=47) | | | | | ***P*** |
|  |  | Genotype 1 (N; %) | Genotype 2 (N; %) | Genotype 3 (N; %) | Allele 1 (N; %) | Allele 2 (N; %) | Genotype 1 (N; %) | Genotype 2 (N; %) | Genotype 3 (N; %) | Allele 1 (N; %) | Allele 2 (N; %) |  |
| rs241141 | A/G | GG (25; 0.42) | AG (28; 0.47) | AA (7; 0.12) | G (78; 0.65) | A (42; 0.35) | GG (20; 0.43) | AG (24; 0.51) | AA (3; 0.06) | G (64; 0.68) | A (30; 0.32) | 0.62 |
| rs8121302 | C/T | TT (15; 0.25) | CT (33; 0.55) | CC (12; 0.20) | T (63; 0.53) | C (57; 0.48) | TT (17; 0.36) | CT (22;0.47) | CC (8; 0.17) | T (56; 0.60) | C (38; 0.40) | 0.29 |
| rs6111826 | G/T | GG (21; 0.35) | GT (32; 0.54) | TT (7; 0.12) | G (74; 0.62) | T (46; 0.38) | GG (14; 0.30) | GT (25; 0.53) | TT (8; 0.17) | G (53; 0.56) | T (41; 0.44) | 0.41 |
| rs761463 | C/T | CC (17; 0.29) | CT (34; 0.57) | TT (9; 0.15) | C (68; 0.57) | T (52; 0.43) | CC (19; 0.40) | CT (24; 0.51) | TT (4; 0.09) | C (62; 0.66) | T (32; 0.34) | 0.14 |
| rs6136363 | A/G | AA (13; 0.26) | AG (29; 0.58) | GG (8; 0.16) | A (55; 0.55) | G (45; 0.45) | AA (14; 0.30) | AG (24; 0.51) | GG (9; 0.19) | A (52; 0.55) | G (42; 0.45) | 0.96 |
| rs13039328 | C/T | CC (21; 0.35) | CT (24; 0.40) | TT (15; 0.25) | C (66; 0.55) | T (54; 0.45) | CC (20; 0.43) | CT (20; 0.43) | TT (7; 0.15) | C (60; 0.64) | T (34; 0.36) | 0.22 |
| rs6045524 | A/T | AA (27; 0.46) | AT (19; 0.32) | TT (13; 0.22) | A (73; 0.62) | T (45; 0.38) | AA (15; 0.32) | AT (25; 0.53) | TT (7; 0.15) | A (55; 0.59) | T (39; 0.41) | 0.64 |
| rs6132097 | C/T | CC (19;0.32) | CT (26; 0.43) | TT (15; 0.25) | C (64; 0.53) | T (56; 0.47) | CC (18; 038) | CT (25; 0.53) | TT (4; 0.09) | C (61; 0.65) | T (33; 0.35) | 0.09 |
| rs6045592 | A/C | CC (16; 0.30) | CA (27; 0.50) | AA (11; 0.20) | C (59; 0.55) | A (49; 0.45) | CC (11; 0.38) | CA (28; 0.60) | AA (8; 0.17) | C (50; 0.53) | A (44; 0.47) | 0.94 |
| rs742731 | A/G | GG (20; 0.36) | AG (27; 0.48) | AA (9; 0.16) | G (67; 0.60) | A (45; 0.40) | GG (17; 0.36) | AG (18; 0.38) | AA (12; 0.26) | G (52; 0.55) | A (42; 0.45) | 0.53 |
| rs6105992 | C/T | CC (23; 0.38) | CT (29; 0.48) | TT (8; 0.13) | C (75; 0.62) | T (45; 0.38) | CC (16; 0.34) | CT (25; 0.53) | TT (6; 0.13) | C (57; 0.61) | T (37; 0.39) | 0.77 |
| rs6045803 | A/C | AA (15; 0.25) | AC (36; 0.60) | CC (9; 0.15) | A (66; 0.55) | C (54; 0.45) | AA (18; 0.38) | AC (17; 0.36) | CC (12; 0.26) | A (53; 0.56) | C (41; 0.44) | 0.84 |
| ^‡^ Utah residents with Northern and Western European ancestry from the CEPH collection. HapMap Data Rel 24/phaseII Nov08 | | | | | | | | | | | | |
| *P*, p value Armitage's trend test | | | | | | | | | | | | |

| **Table IIIs. Haplotypes flanking the E109K mutation** | | | | | | |  | |
| --- | --- | --- | --- | --- | --- | --- | --- | --- |
| **SNP** | **Moroccan-Jewish patients** | | | | | |  | |
|  | 1 | | 2 | | 3 | | | |
| rs241141 A/G | A | A | A | G | G | G | |  |
| rs8121302 C/T | T | T | T | T | C | T | |  |
| rs6111826 G/T | G | G | G | G | G | G | |  |
| rs761463 C/T | C | C | C | C | C | C | |  |
| rs6136363 A/G | G | G | G | G | G | G | |  |
| rs13039328 C/T | C | C | C | C | C | C | |  |
| **c.325 G>A** | **A** | **A** | **A** | **A** | **A** | **A** | |  |
| rs6045524 A/T | A | A | A | A | A | A | |  |
| rs6132097 C/T | T | T | T | T | T | T | |  |
| rs6045592 A/C | A | A | A | A | A | A | |  |
| rs742731 A/G | G | G | G | G | G | G | |  |
| rs6105992 C/T | C | C | C | C | C | C | |  |
| rs6045803 A/C | A | A | A | A | A | C | |  |
| Haplotypes common to European patients are coloured light grey | | | | | | |  | |

**Figure 1s. Families with the R14W mutation**

The pedigrees of 4 Italian families (pedigrees A1-D1) with the R14W mutation are shown. Squares represent males and circles females. Black symbols, represent affected individuals, open symbols, unaffected individuals, gray symbols, affected individuals with a different mutation. Unique patient numbers are shown below the symbols.

**Figure 2s. Families with the E109K mutation**

The pedigrees of 3 Italian families (pedigrees A-C) and 2 NIE families (pedigrees a-b) with the E109K mutation are shown. Squares represent males and circles females. Black symbols, represent affected individuals, open symbols, unaffected individuals, gray symbols, affected individuals with a different mutation. Unique patient numbers are shown below the symbols.


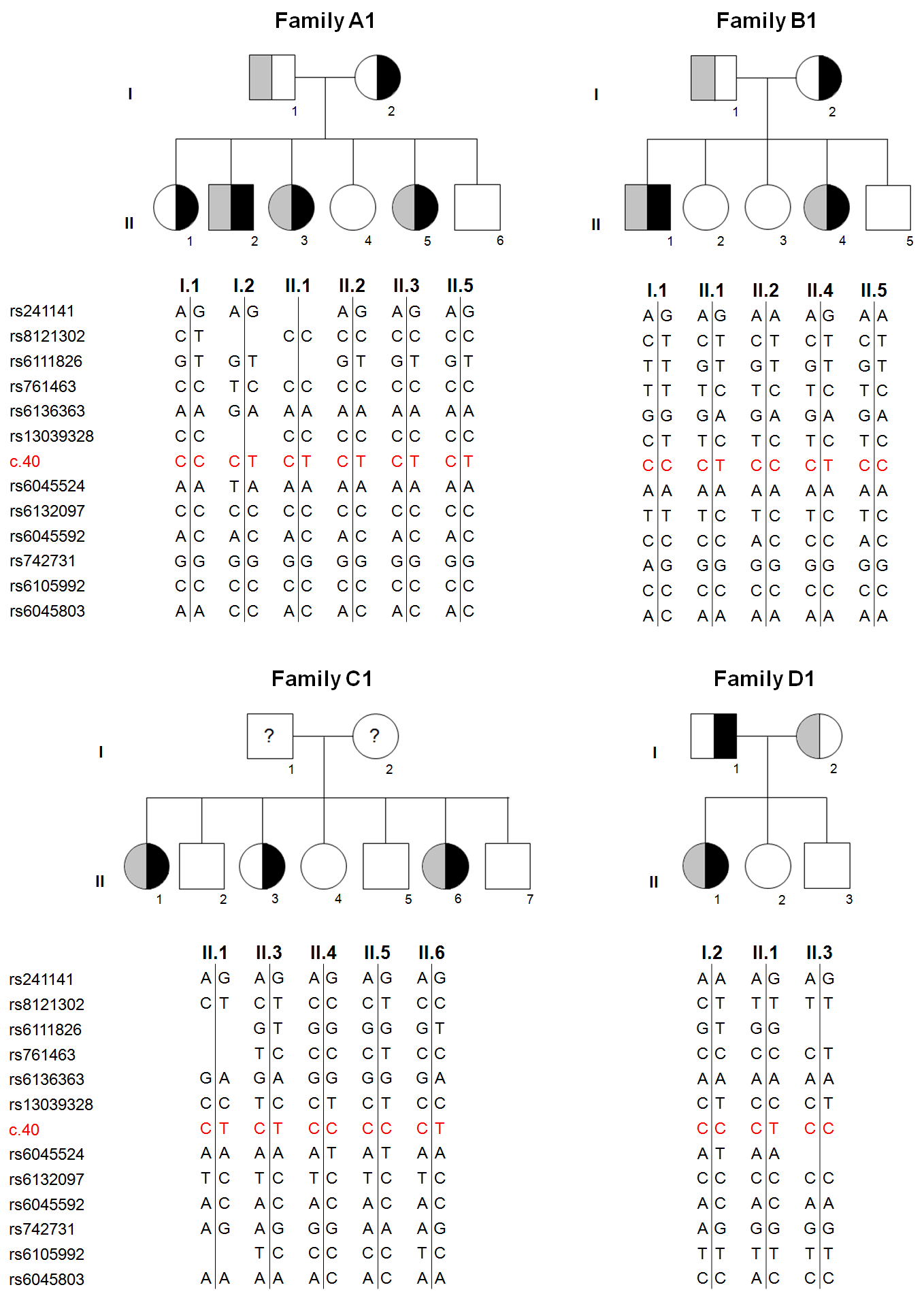


**Figure 1s.**

**
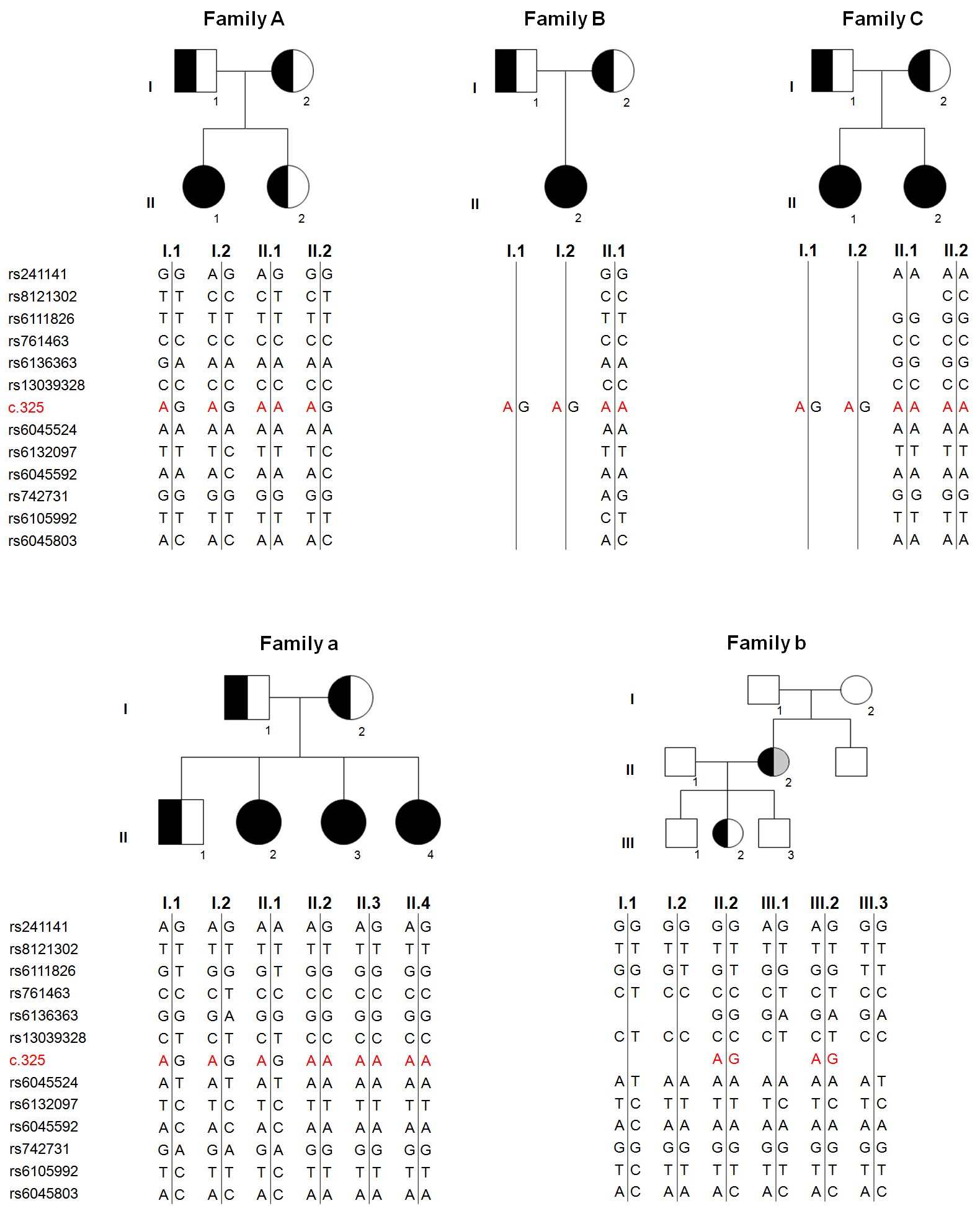
**

**Figure 2s.**
